# Clove Bud Oil Modulates Pathogenicity Phenotypes of the Opportunistic Human Pathogen *Pseudomonas aeruginosa*

**DOI:** 10.1038/s41598-018-19771-7

**Published:** 2018-02-21

**Authors:** Jayalekshmi Haripriyan, Athira Omanakuttan, Nitasha D. Menon, Muralidharan Vanuopadath, Sudarslal Sadasivan Nair, Ross Corriden, Bipin G. Nair, Victor Nizet, Geetha B. Kumar

**Affiliations:** 1 School of Biotechnology, Amrita University, Amrita Vishwa Vidyapeetham, Clappana P.O., Kollam, Kerala 690525 India; 2 Department of Pharmacology, University of California, San Diego, 9500 Gilman Drive, La Jolla, CA 92093 USA; 3 Skaggs School of Pharmacy and Pharmaceutical Sciences, University of California, San Diego, 9500 Gilman Drive, La Jolla, CA 92093 USA; 4 Department of Pediatrics, University of California, San Diego, 9500 Gilman Drive, La Jolla, CA 920093 USA

## Abstract

Earlier studies from our laboratory have demonstrated that clove bud oil (CBO) attenuates expression of certain virulence factors of *Pseudomonas aeruginosa* PAO1. Here, we probe more deeply into the effect of CBO on four pseudomonal proteases - elastase A, elastase B, protease IV and alkaline protease - each known to play key roles in disease pathogenesis. CBO inhibited the activity of these proteases present in the bacterial culture supernatant. Zymography studies indicated that these proteases can activate host matrix metalloproteases (MMPs) to establish infection, through conversion of pro-MMP-2 to active MMP-2. PAO1 is a predominant pathogen in burn wound infections and we show the modulatory effect of CBO on MMPs in an *in vitro* model of burn injury. Furthermore, CBO induced dose-dependent neutrophil extracellular trap formation in human neutrophils. CBO also increased the survival of *C*. *elegans* infected with PAO1, establishing an anti-infective role in a whole animal model of pathogenesis. LC-MS/MS analysis indicated that CBO treatment elicited a significant reduction of signalling molecules (Acyl-Homoserine-Lactone) involved in quorum sensing regulation. Our observations demonstrate that CBO attenuates key virulence mechanisms of this important human pathogen, while concomitantly enhancing host innate immunomodulatory functions, with potential implications for topical therapy against antibiotic-resistant infections.

## Introduction

The looming global crisis of antibiotic resistance, along with the difficulty in treating recalcitrant infections, creates a critical need for new and intelligent therapeutic modalities and novel means to combat pathogens. Strategies that attenuate the virulence of the pathogen, allowing the host immune system to efficiently eliminate the infection, rather than seeking to kill it, could present an effective alternative solution to direct antimicrobial killing. Furthermore, this is also less likely to provide the “life-or-death” selective pressure that drives the evolution of resistance^1^.

*Pseudomonas aeruginosa* is a major cause of secondary infections in immune-compromised patients^2^ and accounts for a significant percentage of total nosocomial infections^3^. *P*. *aeruginosa* possesses both cell-associated and extracellular virulence factors, regulated in a growth phase-dependent manner by well-defined quorum sensing systems^4^. Among these, the Las system uses N-(3-oxododecanoyl)-L-homoserine lactone (3-oxo-C12-HSL) as the signal transducing molecule, whereas the Rhl system employs N butanoyl-L-homoserine lactone (C4-HSL)^5^. *P*. *aeruginosa* also has a species-specific (PQS) signalling system that employs 2-heptyl-3-hydroxy-4-quinolone as the signalling molecule^6^ and contributes significantly to quorum sensing and infection. In these quorum sensing systems, the signalling molecules turn on various virulence determinants by binding to a cognate receptor protein and bringing about a conformational change that in turn exposes DNA binding regions (promoters) upstream of the virulence genes.

*P*. *aeruginosa* causes infections and creates tissue damage and organ system impairment by producing numerous extracellular and cell-associated virulence factors such as proteases, exotoxins, phospholipases, pigments and lipopolysaccharides. Of these, the proteases have been studied extensively for their key impact on toxicity and pathogenicity of the bacteria. Among the four major proteases in *Pseudomonas*, elastase B plays a critical role in the pathophysiological milieu, by activating the mammalian matrix metalloproteinases (MMPs), resulting in extensive ECM degradation and helping to establish infection^7^. Matrix degradation and tissue remodelling are cardinal features of many inflammatory conditions including bacterial infections. Many bacteria employ various means like expression of different ECM-degrading proteases and recruitment of host plasminogen and MMPs to invade the basal lamina and destroy the integrity of the ECM in the connective tissue and interstitial spaces^8^. *P*. *aeruginosa* virulence factors alter airway epithelial cell function and impair wound repair by perturbing the actin cytoskeleton and inducing the activation of epithelial MMP-2^9^. Likewise, MMP-9 was reported to be upregulated six-fold in response to *P*. *aeruginosa* corneal infection^10^.

Phytochemicals and other natural products are often rich repositories of new classes of antibiotics as well as immune-boosting drugs, which together are vital to counter the mounting danger of drug resistance. An attractive approach would be to investigate means by which such natural products could reduce the bacterial virulence and simultaneously alter immunomodulatory properties of the host. For example, recent studies have detailed the mechanistic basis behind the anti-microbial effect of anacardic acid derived from cashew nut shell liquid in direct bacterial inhibition, stimulation of neutrophils to release neutrophil extracellular traps (NETs) that entrap and kill microbes, or inhibition of mammalian MMP-2 and MMP-9 activities^11,12^.

Plant derived essential oils (EO) used in traditional medicine contain biologically active compounds that could play a crucial role in the discovery and development of novel antimicrobial therapeutics^13^. For example, previous reports from our laboratory demonstrates the attenuation of QS-controlled virulence factors of *P*. *aeruginosa* by clove bud oil (CBO)^14^. A compound which can simultaneously modulate bacterial virulence and host factors in a complementary manner for the management of infectious disease cases could attract substantial clinical interest. The present study demonstrates an inhibitory effect of CBO on the secreted virulence factors of *Pseudomonas*, that in turn play a key role in the activation of host MMPs involved in tissue remodelling, to aid in establishment of infection. CBO also induces NET formation, thereby enhancing the innate immunomodulatory function of the host. Furthermore, we extend our studies to an *in vivo* model, employing *Caenorhabditis elegans*, to demonstrate the anti-infective property of CBO in mitigating *Pseudomonas*-driven pathogenicity.

## Results

### 1% CBO has no significant effect on the growth patterns of *P*. *aeruginosa* PAO1

**Figure 1:**
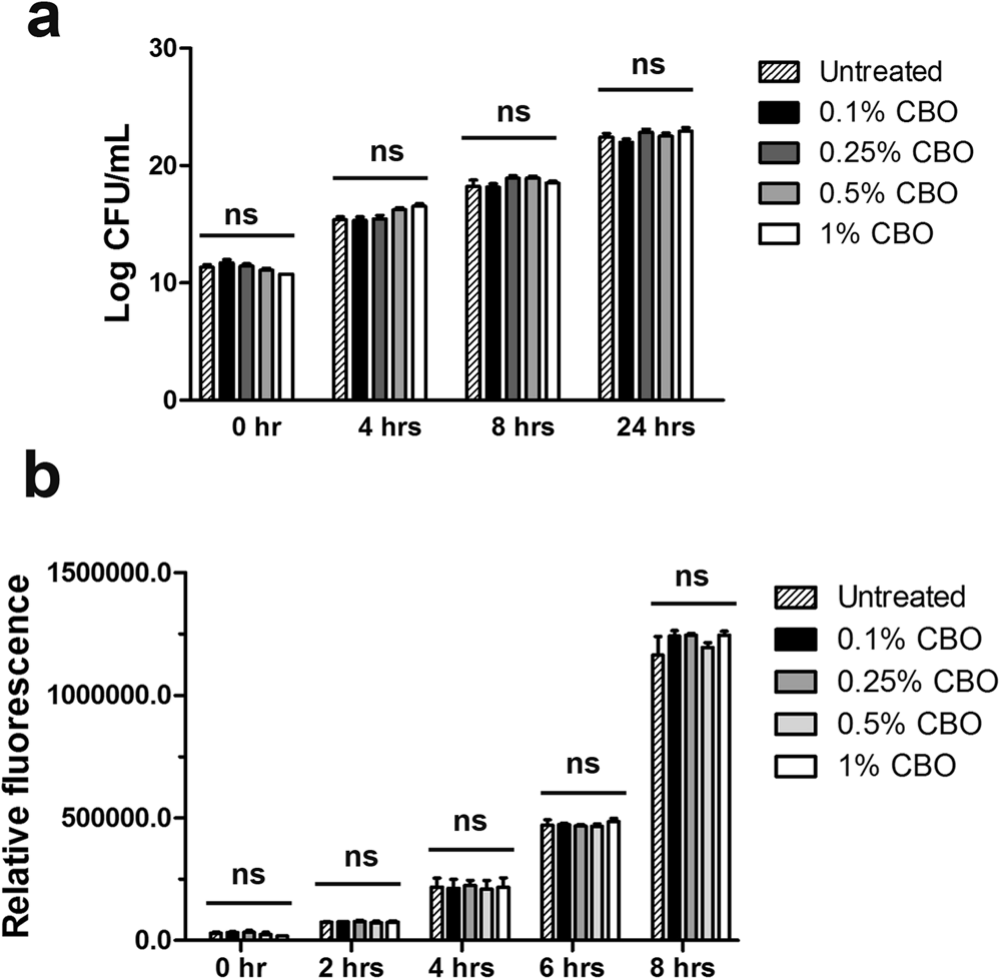
Effect of CBO on *P*. *aeruginosa* growth. (**a**) Cell viability as determined by CFU enumeration and (**b**) cell proliferation as determined by Alamar Blue assay of PAO1 treated with increasing concentrations of CBO. Each value represents mean ± SEM values of three independent experiments. One-way ANOVA followed by Dunnett’s test. ‘ns’ represents not significant.

The viability and cell proliferation of PAO1 as determined by independent methods of CFU enumeration (Fig. 1a) as well as the Alamar Blue assay (Fig. 1b), clearly demonstrate that there is no significant difference in the number of viable cells at all the concentrations of CBO tested when compared to the untreated control.

### CBO inhibits the proteases of *P*. *aeruginosa* involved in virulence

#### Gelatinolytic activities of *P. aeruginosa* culture supernatant (CS)

**Figure 2:**
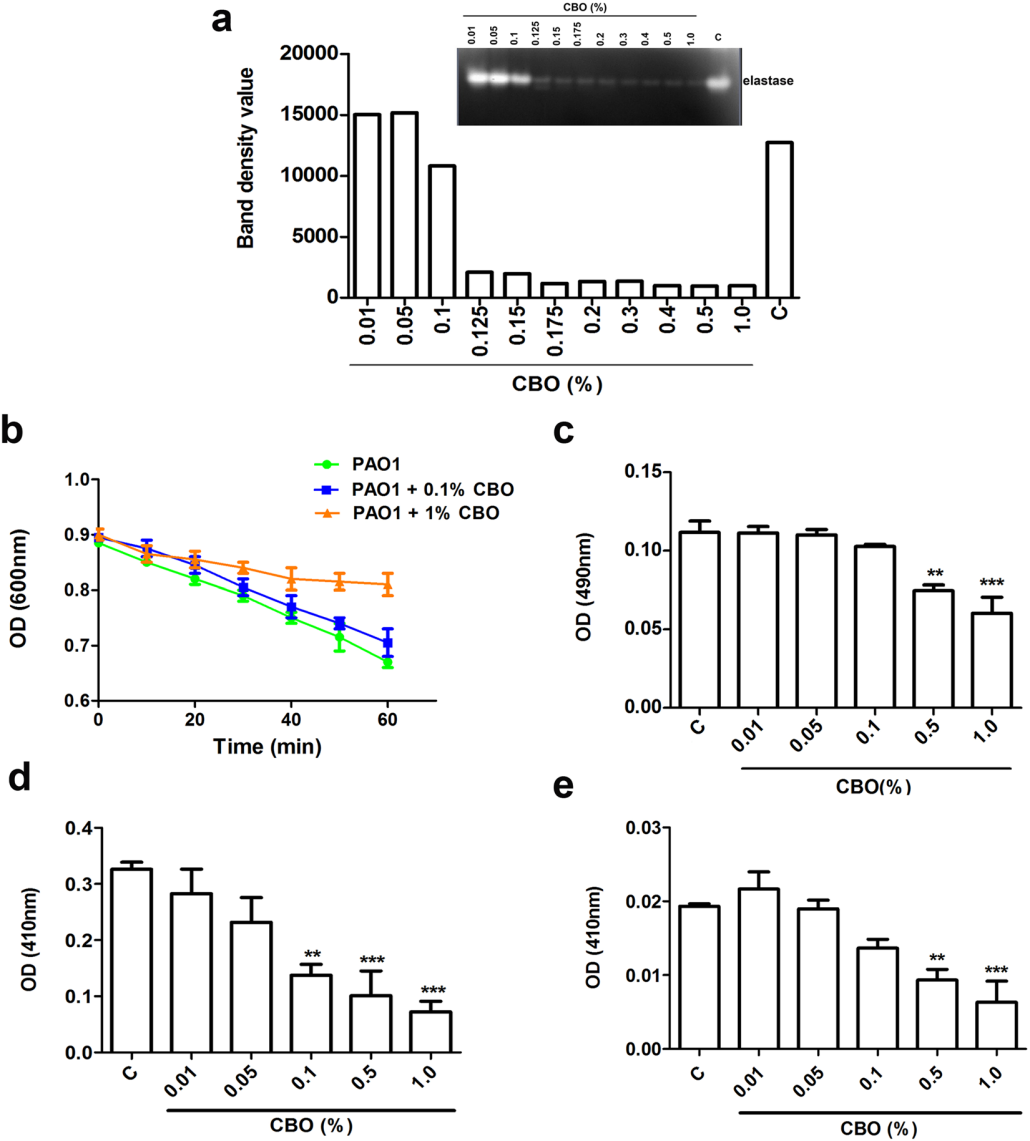
Effect of CBO on *P*. *aeruginosa* protease activities. (**a**) Zymographic analysis (inset) of the dose-dependent effect of CBO on gelatinolytic activity of *P*. *aeruginosa* CS along with a densitometry plot of bands observed in the zymogram. C is the control lacking added CBO. Results shown are representative of three similar experiments. Effect of CBO on *P*. *aeruginosa* (**b**) elastase A (**c**) elastase B (**d**) protease IV and (**e**) alkaline protease. Each value represents mean ± SEM values of triplicate determinations of three independent experiments. Significance at **p < 0.01, ***p < 0.001; One-way ANOVA.

The activities of *P*. *aeruginosa* proteases were visualized by gelatin zymography^15^. The CBO mediated gelatinolytic activity showed that as the concentration of CBO (0.01–1.0%) increases, a dose dependent decrease of gelatinolytic activity was evident from concentrations higher than 0.1% (Fig. 2a). Proteolytic bands with a molecular mass above 200 kDa corresponding to protease IV could not be clearly detected by zymography.

#### Elastase A and elastase B activities

To test the effect of CBO on elastase A, the supernatant of *P*. *aeruginosa* was assayed using a suspension of heat inactivated *Staphylococcus aureus* cells. Elastase A activity was monitored as a rapid decrease in the optical density of the *S*. *aureus* suspension resulting from cell lysis during the 60 minute incubation with the *P*. *aeruginosa* CS. The CBO treatment exhibited a 56% reduction in elastase A activity as compared to the untreated control (Fig. 2b). The activity of elastase B is six-fold greater than that of elastase A, making this protease an important virulence factor during infection. Elastase B activity of *P*. *aeruginosa* treated with 1% CBO was also significantly reduced (by 58.9%) as compared to the untreated control (Fig. 2c).

#### Protease IV and alkaline protease activities

Lysine-specific endoproteases, like protease IV, play an important role in establishing *Pseudomonas*-associated keratitis and pulmonary infections. Cleavage of the chromogenic peptide, Chromozyme PL, by protease IV is specifically inhibited by TLCK (Nα-Tosyl-L-lysine chloromethyl ketone), but not EDTA. Therefore, the specific activites of alkaline protease and protease IV were determined by using EDTA and TLCK respectively with Chromozyme PL as a substrate. The results obtained in the Chromozym PL assays clearly indicated that with increasing concentrations of CBO there was a corresponding decrease in the amount of both protease IV and alkaline protease activity. Treatment with 1% CBO caused 70.8% inhibition of protease IV (Fig. 2d) and 77% inhibition of alkaline protease activity (Fig. 2e).

### *P*. *aeruginosa* CS modulates MMP-2 activity in 3T3L1 fibroblast cells

**Figure 3:**
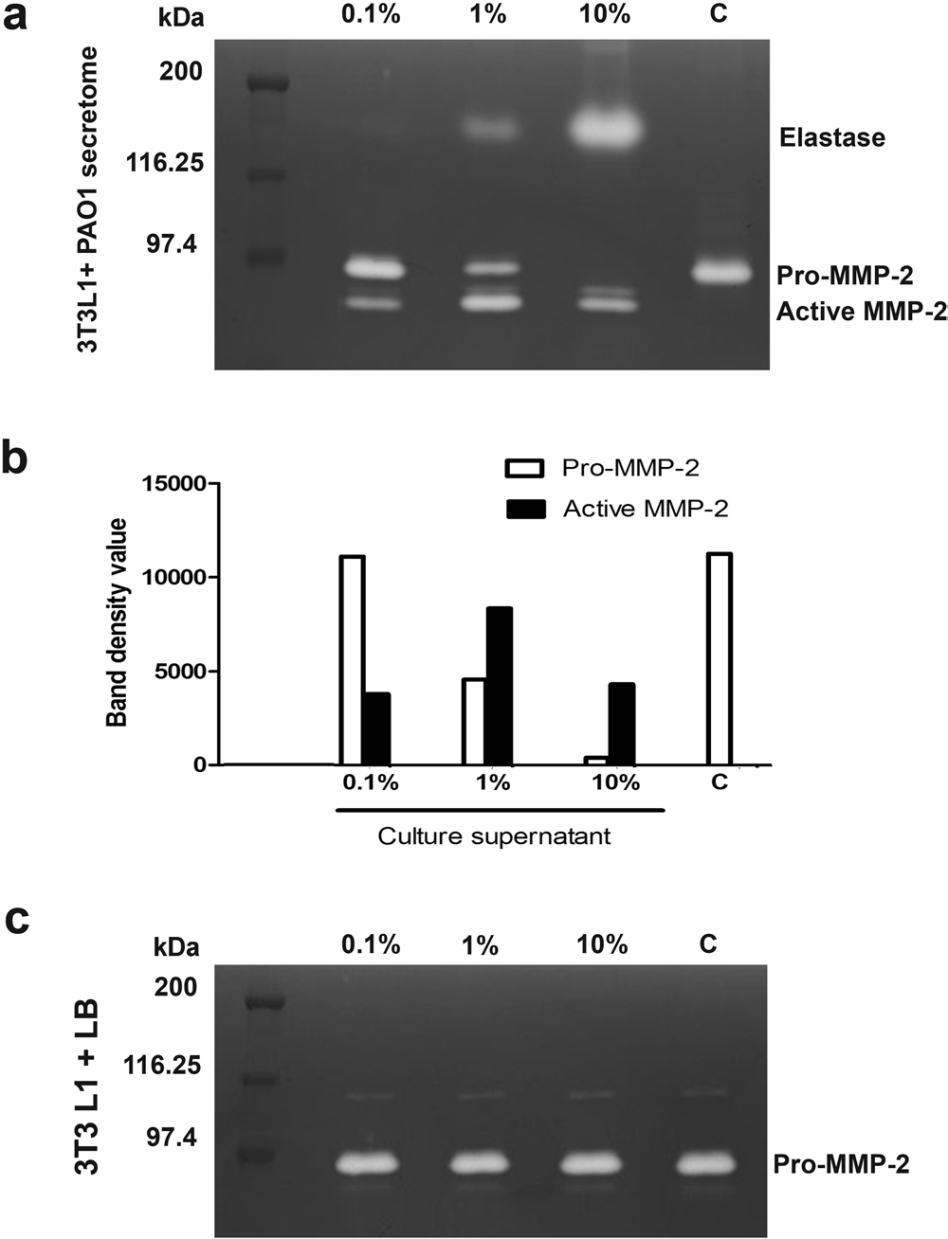
Effect of *P*. *aeruginosa* CS on the gelatinolytic activity of 3T3L1 fibroblast cells. Zymographic analysis of conditioned media from 3T3L1 fibroblast cells treated with (**a**) 0.1%, 1% and 10% dilutions of *P*. *aeruginosa* CS and (**b**) representative densitometry plot of bands observed in the zymogram. (**c**) represents the conditioned media control of 3T3L1 fibroblast cells treated with LB media. Results shown are representative of three similar experiments.

Levels of MMP expression and activation vary considerably from one tissue to another. The present study investigated the activation of pro-MMP-2 in murine fibroblast 3T3L1 cells by *P*. *aeruginosa* CS. Confluent 3T3L1 fibroblast cells were incubated with different dilutions (10%, 1% and 0.1%) of PAO1 CS. After 24 hours, the conditioned media of 3T3L1 cells was collected and zymography was performed. Activation of pro-MMP-2 upon the addition of different concentrations of PAO1 CS is shown in Fig. 3a. Bacterial media (LB) was used as a control to monitor the specificity of the gelatinolytic activation (Fig. 3c).

Addition of 10% of *P*. *aeruginosa* CS to confluent 3T3L1 fibroblast cells resulted in complete processing of pro-MMPs (72 kDa) into active MMPs (62 kDa), whereas in cells exposed to 1% PAO1 CS, the band corresponding to pro-MMP-2 could be also visualised along with the active MMP-2. In the presence of 0.1% PAO1 CS, most of the MMP-2 was present in the pro-form as compared to the active form, clearly establishing a dose dependent activation of the MMP-2 (Fig. 3a). The 3T3L1 cells grown with LB media (0.1%, 1% and 10%) did not show any activation of pro-MMP-2 (Fig. 3b) indicating that the activation was mediated by the CS. Based on these results, further studies were carried out using 1% CS (i.e. 0.1 ml of the CS added to 9.9 ml of DMEM media), since both the pro and the active forms of MMP-2 could be visualized under these conditions (Fig. 3a). These studies indicated that in control 3T3L1 fibroblast cells, MMP-2 was present in its precursor form, whereas in the presence of increasing concentration of PAO1 supernatant, a concomitant increase in the activated form of MMP-2 was observed.

### CBO modulates the activation of pro-MMP-2 in 3T3L1 fibroblast cells on exposure to *P*. *aeruginosa* CS

**Figure 4:**
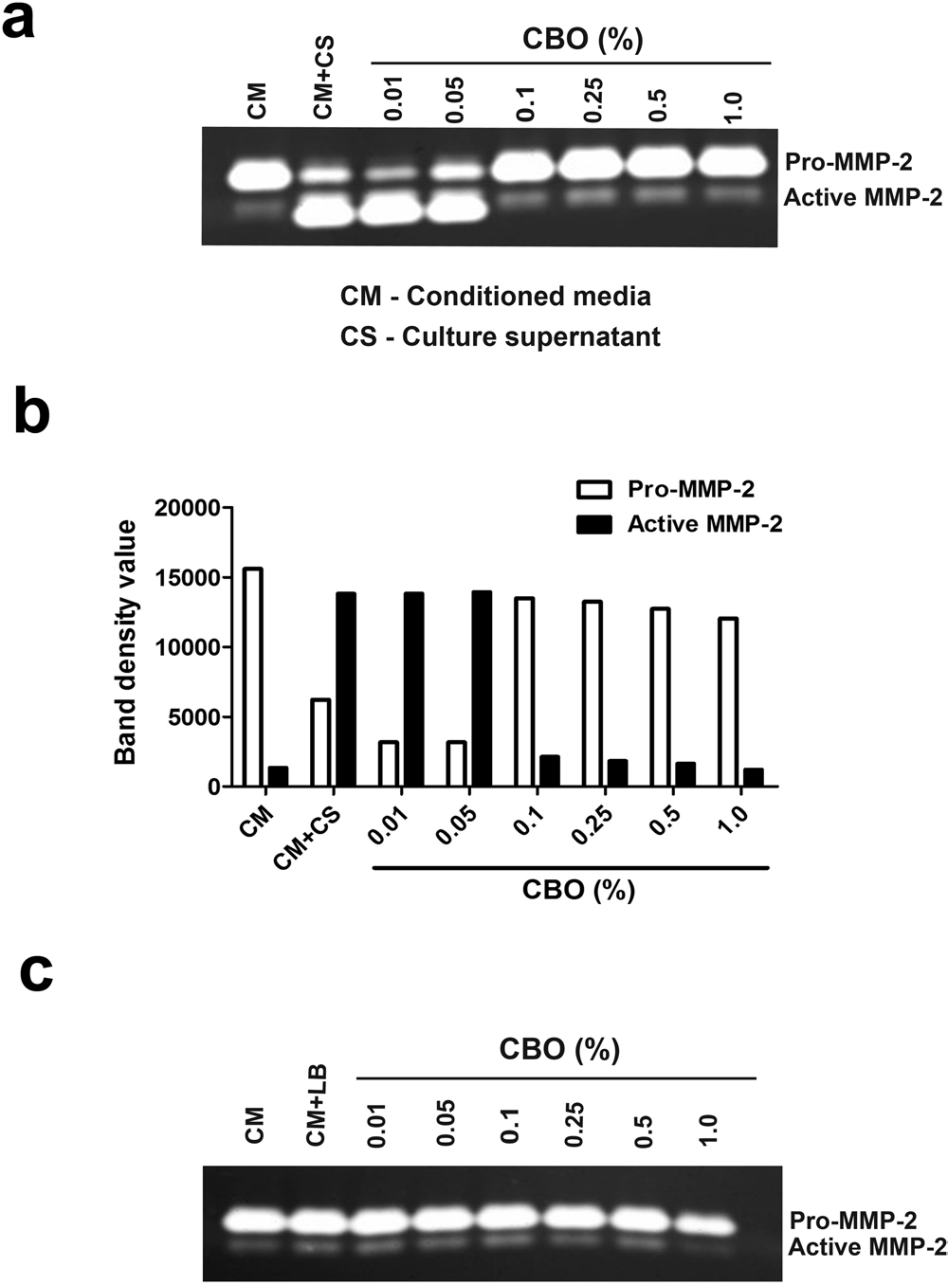
Effect of CBO treated *P*. *aeruginosa* CS on gelatinolytic activity of 3T3L1 fibroblast cells. Zymographic analysis of conditioned media from 3T3L1 cells treated with (**a**) *P*. *aeruginosa* CS with different concentration of CBO and (**b**) representative densitometry plot of bands observed in the zymogram. (**c**) represents the LB media control with different concentrations of CBO. Results shown are representative of three similar experiments.

The modulatory effect of CBO on activation of MMP-2 was investigated by zymography. When the *P*. *aeruginosa* CS (1% v/v, treated with increasing concentrations of CBO) was added to confluent 3T3L1 fibroblast cells, the activation of pro-MMP-2 to MMP-2 was substantially decreased in a dose-dependent manner when compared to the control cells (Fig. 4). These results support our earlier studies which demonstrated that CBO inhibited the elastase of *P*. *aeruginosa*, presumed to be responsible for the activation of pro-MMP to activated MMP-2.

### CBO inhibits maturation of MMP-2 during wound repair in 3T3L1 fibroblast cells

**Figure 5:**
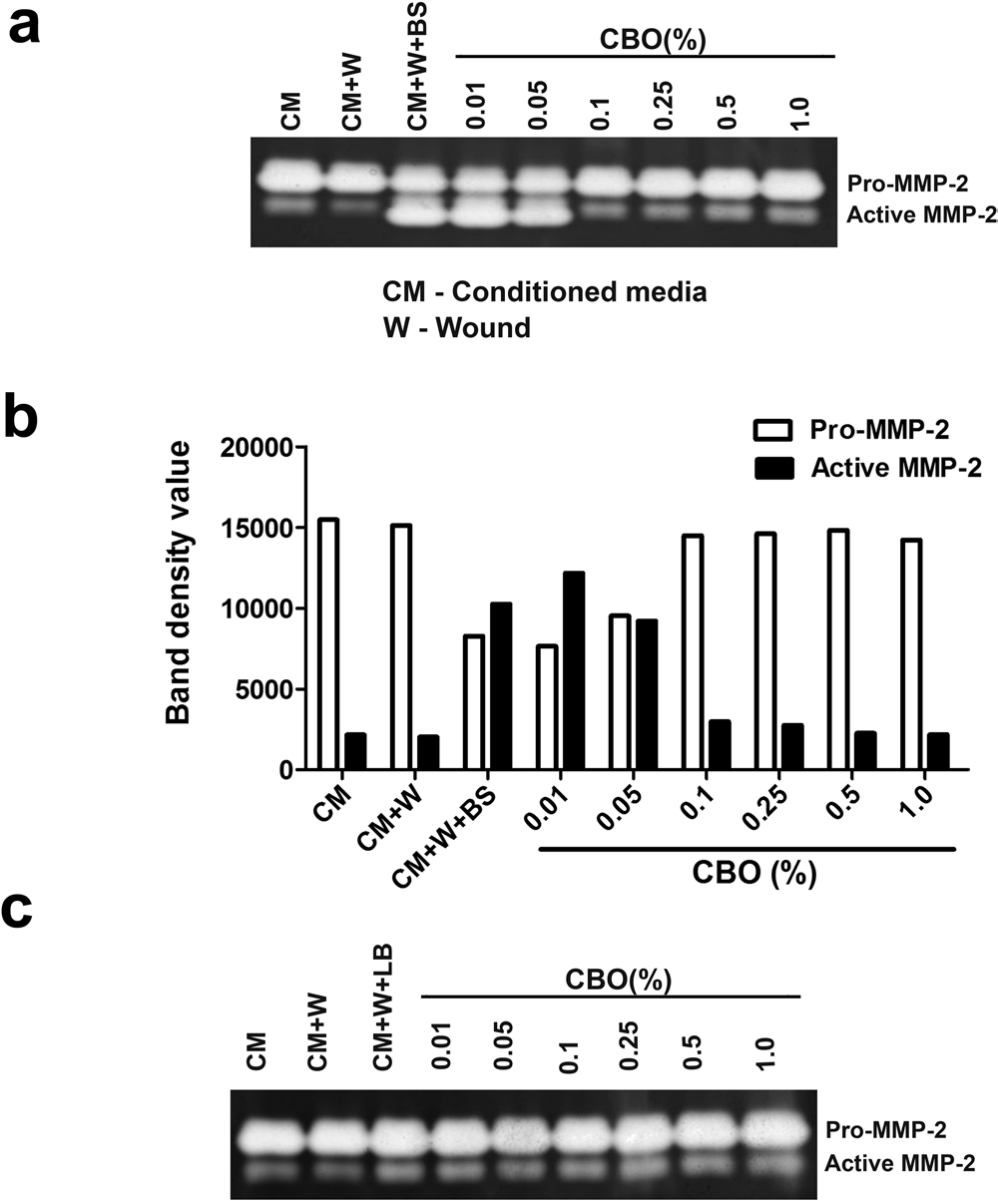
Effect of CBO treated *P*. *aeruginosa* CS on gelatinolytic activity of 3T3L1 fibroblast cells wounded with 0.1 N NaOH. (**a**) Zymographic analysis of gelatinolytic activity of conditioned media from 3T3L1 fibroblast cells treated with CS of *P*. *aeruginosa* exposed to varying concentrations of CBO along with (**b**) representative densitometry plot of bands observed in the zymogram. (**c**) LB media control with different concentrations of CBO. Results shown are representative of three similar experiments.

Secondary infections of burn wounds by *P*. *aeruginosa* are a major concern in clinical settings. We were therefore interested in understanding the effect of CBO on the CS-mediated activation of MMP-2 during wound repair. 3T3L1 cells were chemically injured by the addition of 0.1 N NaOH. After 24 hours of wound induction, *P*. *aeruginosa* CS (1% v/v), previously treated with increasing concentration of CBO was added and the gelatinase activity detected by zymography. As shown in Fig. 5, with increasing concentration of CBO there was a corresponding decrease in the activation of pro-MMP-2 to active MMP-2. These observations indicate that CBO controls induction of pro-MMP-2 to active MMP-2 in an *in vitro* model of burn injury, an important factor for establishment of PAO1 infections.

### CBO stimulates production of neutrophil extracellular traps

**Figure 6:**
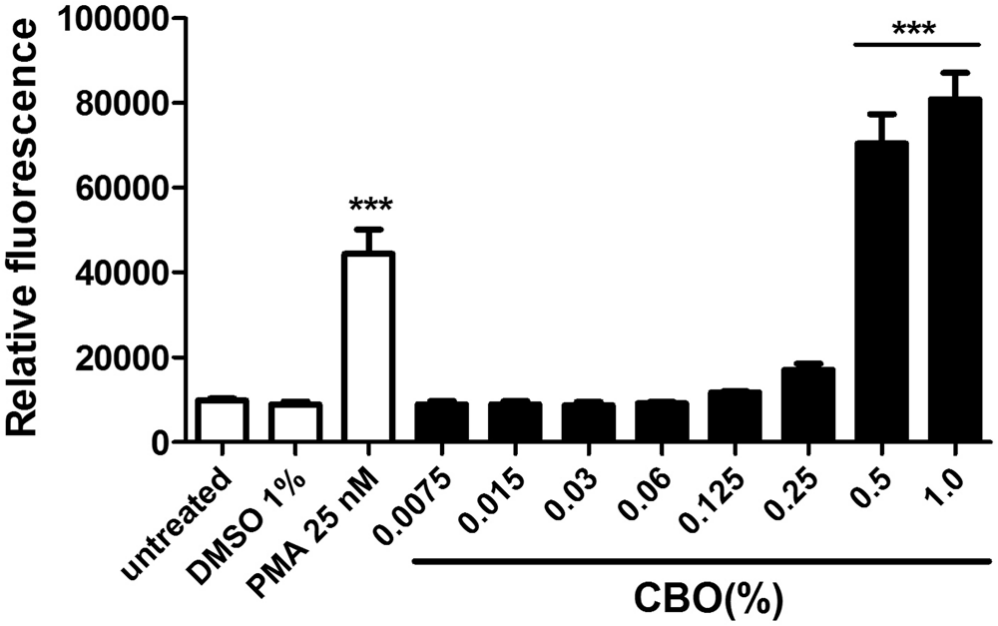
CBO induced NET formation by human neutrophils. NET quantification was done after treatment of neutrophils with varying concentrations of CBO, 25 nM PMA (positive control), and 1% DMSO (negative control) for 2.5 hours. Each value represents mean ± SEM of three determinations from three different experiments compared to control of untreated cells. ***p < 0.001; One-way ANOVA.

Neutrophils are an important front line of defence against pathogenic bacterial infection, and one key pathway for bacterial killing by these cells is the elaboration of neutrophil extracellular traps (NETs). NETs, which are generated through a specialized cell death process, are comprised of a DNA backbone upon which antimicrobial peptides, histones and myeloperoxidase are deployed, and function to ensnare and eradicate bacteria^16,17^. Neutrophils respond to *P*. *aeruginosa* by releasing NETs, which are a major contributor to killing of non-mucoid strains^18,19^. We found that CBO at concentrations greater than 0.5% induced robust dose-dependent production of NETs by freshly-isolated human neutrophils (Fig. 6), suggesting an innate immune boosting property that may synergize with the virulence blocking properties of the natural compound to aid in treatment of *P*. *aeruginosa* infection.

### CBO enhances the survival of *C*. *elegans* through the attenuation of QS in *P*. *aeruginosa*

**Figure 7:**
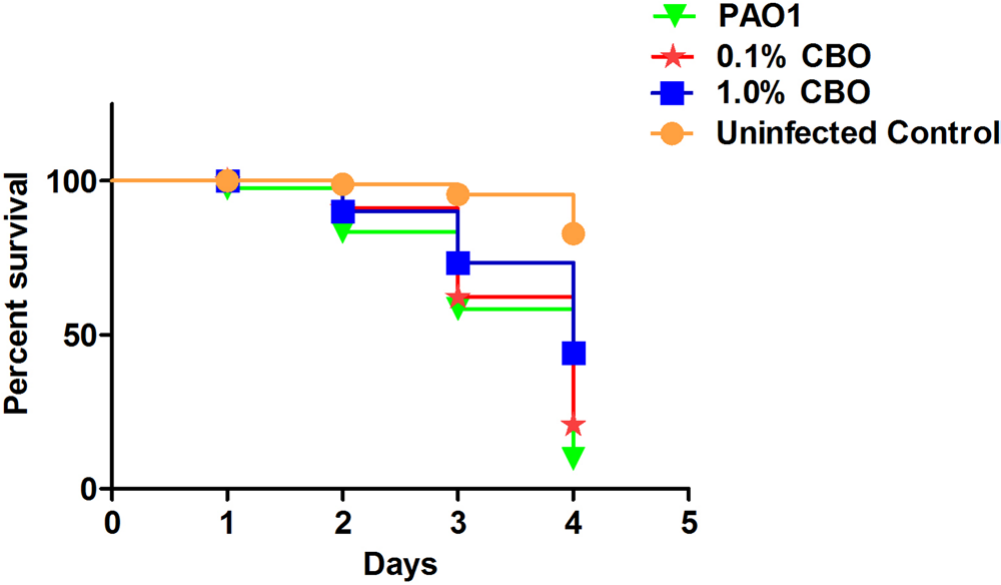
Survival analysis of *C*. *elegans* grown with CBO treated/untreated *P*. *aeruginosa* cells. Results shown are representative of three seperate experiments.

*C*. *elegans* is recognized as a relevant model for studying bacterial virulence^20–22^. *C*. *elegans* was infected with PAO1 treated with different concentration of CBO and survival was compared to untreated controls. On average, the survival rate of the worms infected with untreated PAO1 was only 13.9% between day 1 and 4 while over 50% of the worms infected with 1% CBO treated PAO1 were alive at the 4-day mark. (Fig. 7). The enhanced survival of worms infected with CBO-treated *Pseudomonas* demonstrated the capacity of CBO to attenuate the virulence of PAO1 and provides inspiration for further investigations of its potential use as an anti-infective agent.

### CBO regulates AHLs of *P*. *aeruginosa*

**Figure 8:**
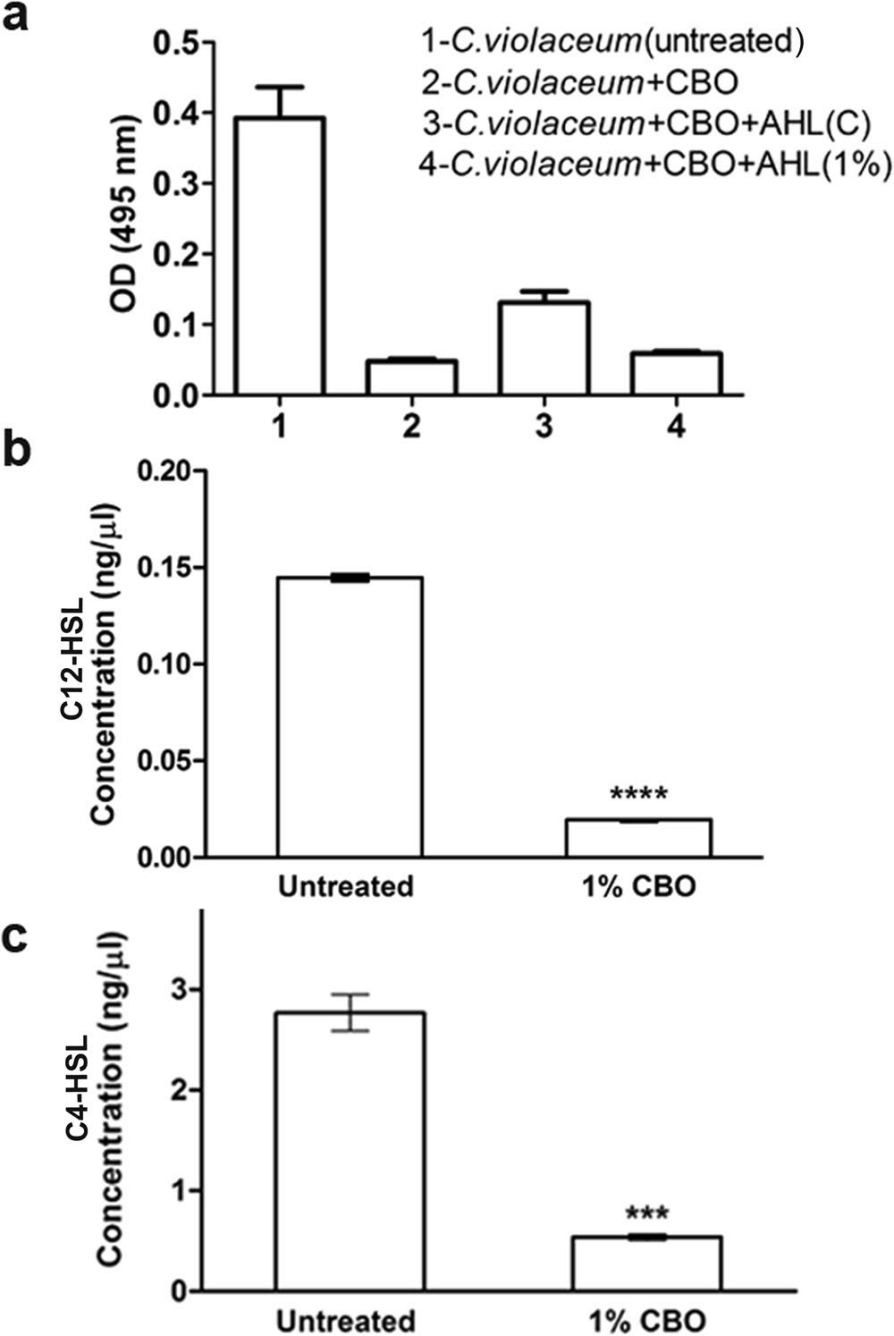
(**a**) Effect of CBO on AHL of *P*. *aeruginosa*, by using the biosensor strain *C*. *violaceum* 12472. *C*. *violaceum* + CBO + AHL(C) refers to Acyl Homoserine Lactone (AHL) extracted from untreated *Pseudomonas* (control) and added to *C*. *violaceum* pre-treated with 0.1% CBO. *C*. *violaceum* + CBO + AHL (1%) refers to Acyl Homoserine Lactone (AHL) extracted from *P*. *aeruginosa* treated with 1% CBO and added to *C*. *violaceum* pre-treated with 0.1% CBO. LC-MS/MS based quantification of (**b**) C12-HSL and (**c**) C4-HSL of *P*. *aeruginosa* treated with 1% CBO. Each value represents mean ± SD values of triplicates. ****p < 0.0001; ***p < 0.001; Student’s t-test.

To check the effect of CBO on signalling molecules (AHLs) of *P*. *aeruginosa*, we used the biosensor strain *C*. *violaceum* 12472 which responds to external QS signalling molecules. The violacein pigment production which is controlled by QS was dramatically reduced in the presence of CBO (Fig. 8a). Furthermore, a considerable increase in violacein production (2.6 fold) occurred when AHLs extracted from untreated *P*. *aeruginosa* were added. However, when the AHLs extracted from *P*. *aeruginosa* cultures treated with 1% CBO were added, violacein production was significantly decreased (Fig. 8a), indicating a reduction in the AHL signalling molecule. These results were further confirmed by subjecting the AHL extracts obtained from *P*. *aeruginosa* treated with 1% CBO using LC-MS/MS analysis and compared with untreated controls. The concentration of C12-HSL and C4-HSL extracted from supernatants were determined using the appropriate standards. As shown in Fig. 8b and c, there was a substantial decrease in C12-HSL (7 fold) and C4-HSL (5 fold) from *P*. *aeruginosa* cultures treated with 1% CBO as compared to the untreated control. Taken together, these observations clearly indicate that CBO regulates the AHLs of *Pseudomonas*, which are involved in virulence.

## Discussion

Plant derived essential oils (EO) are bountiful reservoirs of biologically active compounds traditionally used as medicines. Earlier studies from our laboratory had established that 1% CBO attenuated the virulence factors of PAO1 without exhibiting any bactericidal effects^14^. The present study expands considerably on the results of our earlier observations, to demonstrate that CBO at sub-inhibitory concentrations (1%) attenuates the virulence of *P*. *aeruginosa* PAO1 as well as influences a variety of host immunomodulatory functions. The cell viability and cell proliferation of PAO1 are not significantly affected in the presence of CBO as demonstrated by the CFU enumeration and the Alamar Blue assay, respectively. Furthermore, other published studies also indicate that the MIC of CBO for PAO1 is well above 5%^23^. Therefore the inhibition of bacterial proteases, mammalian metalloproteases, enhanced NET formation and decrease in levels of signalling molecules as well as the enhanced survival of *C*. *elegans* exhibited in the whole animal model of pathogenesis are CBO specific.

The ability of secreted virulence factors of pathogenic bacteria to alter the host responses is well established. However, earlier studies have mainly focused on toxins that are transported into the cytoplasm or taken up by the target cells^24^. *P*. *aeruginosa* is a notorious opportunistic pathogen responsible for a wide variety of clinically important infections. The severity and the magnitude of its pathogenicity are mediated by host-pathogen interactions, which in turn are determined by the virulence factors of the pathogen and their immunomodulatory effects on host defence mechanisms. *P*. *aeruginosa* possesses an arsenal of cell-associated and secreted virulence factors that play a key role in establishing and spreading infection. Of the secreted factors, elastase A, elastase B, protease IV and alkaline proteases are considered among the most potent virulence determinants^25^. Alkaline protease is involved in invasion and haemorrhagic tissue necrosis, and is also implicated in inhibition of phagocytosis by human neutrophils. Biologically important proteins like immunoglobins, fibrinogen, plasminogen, and complement components can be digested by protease IV and play an important role in establishing keratitis^26^. The present study demonstrates that CBO significantly reduces the activity of both alkaline protease and protease IV. Hence, the infectivity and the immunomodulatory properties of CBO-treated *Pseudomonas* would most likely be significantly altered when compared to the untreated pathogen.

Our observations further established that CBO treatment results in inhibition of both elastase A and B of *P*. *aeruginosa*, two metalloproteases that also play an important role in pathogenesis. *P*. *aeruginosa* has developed a defence strategy against the commonly competing microbe *S*. *aureus*, through elastase A (staphylolysin) expression, to kill the competitor species and help in colonization of the pathogen^27^. Elastase B, encoded by *lasB*, plays a key role in establishing pulmonary cystic fibrosis and corneal infections and prevalence of *lasB* expression is high among clinical isolates. *P*. *aeruginosa* strains deficient in elastase B are significantly less virulent in inducing infections^28^.

Proteins from wound fluids and human skin biopsies (*ex vivo*) are degraded by strains of *P*. *aeruginosa* that produce elastase B^29^. Hence, the invasiveness of *P*. *aeruginosa* in patients with burn wounds correlates with elastase B production^30^. Elastase B is also involved in degradation of the tight junctions between epithelial cells^31^ and in enhancing interleukin-8 (IL-8) production by activation of the mitogen-activated protein kinase (MAPK) pathway via extracellular signal-regulated (ERK1/2) proteins^32^. All of these studies provide concrete evidence that elastase B production by *P*. *aeruginosa* is a pivotal strategy used by the bacteria to counteract complex host defence mechanisms. Since our studies indicate that the elastase B activity of *P*. *aeruginosa* treated with 1% CBO was significantly reduced compared to the untreated control, it is likely that CBO-mediated inhibition of this protease could attenuate the virulence and aid in protection against *P*. *aeruginosa* infections. Lastly, elastase B plays a key role in activating the mammalian matrix metalloproteases that break down host collagen type III and IV and helps in establishing infection^9^. Results obtained from our zymography studies support these observations by demonstrating a dose-dependent decrease in the gelatinolytic activity.

Although *P*. *aeruginosa* is a prominent pathogen responsible for severe infections, the direct effect of secreted proteases of *P*. *aeruginosa* on fibroblast cells has not been studied. Elastase B appeared to be the predominant factor responsible for activation of MMP-2, involved in tissue destruction and establishment of infection. The supernatants obtained from elastase B-deficient PDO 240 strain and another Gram-negative bacterium (*E*. *coli*) did not exhibit this effect^9^. We found that secreted *P*. *aeruginosa* elastase processed pro-MMP-2 to its active form. In control 3T3L1 fibroblast cells, the MMP-2 was present in its precursor form, and upon treatment with increasing concentration of PAO1 supernatant, the activated form of MMP-2 becomes predominant. An inhibitory effect of CBO on elastase was reflected in the dose-dependent decrease in activation of pro-MMP2 to active MMP-2.

Burn wounds are complex micro-environments where infection by bacteria such as *P*. *aeruginosa* prevents healing and instigates disease progression. Damage incurred due to burns can be manifested in different levels, ranging from skin to underlying tissues^33^. A balance between host proteinases and their inhibitors is involved in tissue remodelling and wound repair. MMPs are important proteinases that digest extracellular material and allow an influx of restorative keratinocytes, fibroblasts and endothelial cells^34^. In chronic wounds the balance between MMPs and its regulators are altered. Trengove *et al*. found elevated levels of MMP-2 and MMP-9 in non-healing chronic wounds in response to bacterial infections^35^. Our findings on the effect of *P*. *aeruginosa* CS on 3T3L1 fibroblast cells showed that MMP-2 levels were significantly increased, which may lead to more matrix destruction and dissemination of infection. CBO exhibited a regulatory effect on CS-mediated activation of MMP-2 in chemically wounded 3T3L1 fibroblast cells, providing a new rationale for CBO-mediated control of *Pseudomonas* infections.

The proteases of *P*. *aeruginosa* are regulated by Las and Rhl QS systems which are at the apex of the quorum sensing hierarchy^36^. The signalling molecules, C12-AHL and C4-AHL, which correspond to Las and Rhl QS system respectively, are known to mediate virulence as well as exert immunomodulatory effects. In the presence of CBO, we observed impairment in the levels of C12-AHL and C4-AHL, which correspond to Las and Rhl QS system respectively. Reduced levels of AHLs by CBO might interfere with the normal secretion of PAO1 virulence factors such as proteases. These results were further supported by the CBO-mediated inhibition of the violacein pigment production by *C*. *violaceum* 12472 employing the anti-QS colorimetric assay. These observations indicate that the anti-virulence capacity of CBO may be augmented by its interference with the Las and Rhl-dependent quorum sensing pathways.

In addition to attenuating the virulence of *P*. *aeruginosa*, CBO also induces the immunomodulatory function of the host. Neutrophils, the first line of defence in the immune system, can eliminate bacteria through multiple mechanisms including NET formation, phagocytosis, granule release and ROS production^37^. NETs are composed primarily of DNA, histones, and proteins from neutrophil granules and play an effective role in the eradication of various bacterial pathogens by degrading virulence factors and/or killing bacteria extracellularly^38^. The results from the NET quantification assay demonstrate that CBO induces a dose dependent production of NETs, to levels even higher than that produced by PMA, a known inducer of NET formation^39^.

The anti-virulence property of CBO was further corroborated by using *C*. *elegans*, an established *in vivo* invertebrate model, for studying host-microbe interactions^40^. We assessed survival of nematodes infected with *P*. *aeruginosa* and treated with CBO compared to untreated controls. Pre-treatment of PAO1 with CBO considerably reduced the bacterial pathogenicity, resulting in an increase in the survival rate of *C*. *elegans*. Death of *C*. *elegans* mediated by *P*. *aeruginosa* PAO1 is due to cyanide asphyxiation and paralysis^41^. Quorum sensing regulators like Las, Rhl, and PQS control the *hcn* operon mediated cyanide production in *P*. *aeruginosa*^42^. These results suggest that the addition of CBO has a direct (through *hcn*), or indirect (mediated by QS genes), irreversible effect on the production of cyanide, during the time frame of these experiments. Our previous studies, which demonstrated a significant reduction of pyocyanin production and *pqsA* gene expression in *P. aeruginosa*^14^, seems to support the latter hypothesis. Additionally, the experiments detailed in Fig. 8 reveal a significant decrease in the Las (C12-HSL) and Rhl (C4-HSL) signalling molecules, which regulate pseudomonal cyanide production through the hcn operon. The enhanced survival of worms infected with CBO-treated *P. aeruginosa* therefore demonstrates the capacity of CBO to attenuate the virulence of PAO1 in a whole animal model of pathogenesis, and merits further exploration as an anti-infective agent.

Thus, the present study establishes an innate immune boosting property exhibited by CBO, which supports its anti-virulence capacity and thus provides a two-pronged approach by which CBO inhibits *P*. *aeruginosa* infections. Since there is extensive experience in which CBO has been used in traditional Chinese and Indian medicine as a topical remedy for toothaches^43,44^, it is intriguing to contemplate its repurposing for the topical treatment of localized infections (e.g. otitis externa, spa pool folliculitis, ecthyma gangrenosum, chronic leg ulcers) caused by antibiotic-resistant strains of *P*. *aeruginosa*.

## Methods

### Bacterial strains and treatments

*P*. *aeruginosa* wild type strain, PAO1 (ATCC 15692), *Staphylococcus aureus* (ATCC 9144) and *Chromobacterium violaceum* (ATCC 12472) were obtained from American Type Culture Collection, Manassas, VA, USA and propagated in Luria Bertani (LB) broth at 37 °C. For key experiments, bacteria were treated with commercially obtained CBO (*Syzygium aromaticum*) (Plant Lipids Pvt. Ltd., Cochin, Kerala, India) for 24 hours prior to analysis.

### Preparation of bacterial CS

Bacteria were cultured overnight at 37 °C with shaking at 100 rpm in LB liquid medium, until the cells reached stationary phase. Sterile, bacterium-free CS were centrifuged at 10,000 × *g* for 10 min, filtered through a 0.22 µm membrane filter, and checked for their sterility by plating on LB agar, before being stored at −80 °C in 1 ml aliquots^25^.

### Cell viability and proliferation assays

#### Colony Forming Unit (CFU) Enumeration

Cell viability was assessed by CFU enumeration according to the method of Tyc *et al*.^45^. Briefly, aliquots of bacterial cultures were taken at specified time points and serially diluted in sterile PBS and spread on LB agar plates, which were incubated for 24 hours at 37 °C. Viable colonies were counted to determine the CFU/mL.

#### Alamar Blue assay for assessing cell proliferation

The Alamar Blue Resazurin based assay for determining cell proliferation was carried out as described by Tyc *et al*.^45^. Briefly, at each time point, 20 µl of Alamar Blue Resazurin (1 mM) was added to 180 μl of bacterial culture and incubated at 37 °C for 30 minutes. Color change (blue to pink) due to reduction of non-fluorescent resazurin to pink resorufin by the bacterial metabolic activity was recorded. The fluorescence was measured using the BioTek Synergy™ HT Multi-Mode Microplate Reader with excitation/emission wavelengths of 530 nm and 590 nm respectively.

### Assays for the proteases of *P*. *aeruginosa*

#### Elastase A activity (Staphylolytic assay)

For determining the staphylolytic activity of the *P*. *aeruginosa* CS, *S*. *aureus* was cultured for 16–18 hours followed by centrifugation at 10,000 rpm for 5 minutes at 4 °C. The supernatant was discarded, the pellet suspended in 0.02 M Tris-HCl (pH 8.5), and the suspension boiled for 10 minutes and diluted with the same buffer to an optical density of 0.8 at 595 nm. A 100 μl aliquot of the CS was added to 900 μl of boiled *S*. *aureus* suspension. The OD_600_ was determined every 5 minutes for the duration of 1 h. The rate of decrease in absorbance caused by cell lysis was monitored using a plate reader^15^.

#### Elastase B activity (Elastin Congo Red assay)

The elastolytic activity of *P*. *aeruginosa* was determined as previously described^15^ using Elastin Congo Red (ECR, Sigma) as the substrate.

#### Alkaline protease and Protease IV activity (Colorimetric Peptide Assay)

Alkaline protease and protease IV from the *P*. *aeruginosa* CS were examined for the ability to hydrolyze the chromogenic peptide, Chromozym PL (tosyl-Gly-Pro-Lys-p-nitroanilide, Sigma). As described previously^15^,10 μl of the CS was mixed in a microtiter plate with 20 μl of Chromozym PL substrate (2 mg/ml), along with 90 μl of reaction buffer consisting of 50 mM Tris–HCl (pH 8.0) + 150 mM NaCl, in the presence of 100 mM EDTA or 1 mM TLCK (Tosyl-lysine Chloro-methyl Ketone, Sigma). The degradation assay was performed specifically for alkaline protease or protease IV by addition of EDTA (inactivates alkaline protease) or TLCK (which inactivates protease IV). After 30 minutes of incubation at 37 °C, the optical density was read at 410 nm^15^.

### Cell culture

3T3L1 mouse fibroblast cells were obtained from ATCC through the National Centre for Cell Sciences, Pune, Maharashtra, India. The cells were cultured in Dulbecco’s Modified Eagle Medium (DMEM) with 1% penicillin, 1% streptomycin and 0.1% amphotericin B (Sigma Aldrich, St Louis, MO)^11^. 3T3L1 cells were seeded in 24-well plates and upon reaching confluency were washed twice with phosphate buffered saline (PBS) and subsequently exposed to 0.1–10.0% of either the bacterial CS or the untreated control LB medium.

### Collection of cell culture conditioned media

Following exposure to bacterial CS and incubation for 24 h, the conditioned media was collected, and the detached floating cells were pelleted by centrifugation at 2,000 × *g* for 5 min. The cell free media was further centrifuged at 12,000 × *g* for 5 minutes at 4 °C to remove any debris and the supernatant was stored at −20 °C for further analysis.

### *In vitro* fibroblast wound induction

After 24 hours of growth and upon reaching confluency, the culture medium was aspirated. The fibroblast cell monolayer was injured by adding 1 µl of 1 N NaOH dropwise into the centre of the culture dish and immediately washing twice with sterile PBS. The cells that had been in contact with NaOH desquamate and create a circular wound. The wounded culture was then rinsed and incubated at 37 °C with DMEM. After 24 hours of injury induction, *P*. *aeruginosa* CS or sterile LB control were added to the medium and the cells incubated at 37 °C for 24 h, at which time conditioned media was collected. The collected media was centrifuged to remove debris and stored at −20 °C until further analysis. Experimental dilution of the CS or the LB control is expressed as the percentage of the volume of bacterial culture medium in the final reaction volume^9^.

### Neutrophil extracellular trap (NET) quantification assays

Human venous blood was drawn from healthy volunteers under written informed consent, with heparin added as an anticoagulant, according to a protocol approved by the UCSD Human Research Protection Program/IRB. Polymorphprep (Axis Shield, Dundee, Scotland) was used to isolate neutrophils according to the manufacturer’s instructions and neutrophil pellets were resuspended in HBSS (with Ca^2+^/Mg^2+^) at a concentration of 2 × 10^6^cells/ml and added to 96-well plates (2 × 10^5^ cells/well). HBSS and various concentrations of either CBO or phorbol 12-myristate 13-acetate (PMA) were added to applicable wells to a final volume of 200 μl. Following 2.5 hours of incubation at 37 °C with 5% CO_2_, 50 milliunits of micrococcal nuclease (in a volume of 50 μl) was added to each well. After 10 minutes of incubation at 37 °C, EDTA was added to each well (final concentration, 5 mM) to stop the nuclease reaction. The plates were then centrifuged for 8 minutes at 200 × *g*, and 100-μl supernatant samples were collected and transferred to a flat-bottomed 96-well assay plate. DNA was quantified using PicoGreen DNA dye (Life Technologies) and a SpectraMax M3 plate reader (Molecular Devices) as per the manufacturers’ instructions^12^.

### Gelatin zymography

The zymography assay was performed according to the protocol previously described^46^.

### *Caenorhabditis elegans* Survival Assay

The ability of CBO-treated *P*. *aeruginosa* PAO1 to infect *C*. *elegans* was evaluated via slow killing in liquid media^47^. *C*. *elegans* was grown in nematode growth media agar (NGM-Peptone 2.5 g/L, NaCl 2.9 g/L, Bacto-Agar 17 g/L, CaCl_2_ 1 mM, cholesterol 5 μg/mL, KH_2_PO_4_ 25 mM, MgSO_4_ 1 mM) pre-seeded with *E*. *coli* OP50. Nematodes were synchronised by harvesting the eggs from gravid adults (by exposure to 3.75% hypochlorite and 1 N sodium hydroxide) and transferred to a 96-well plate with 89% M9 buffer (0.3% monobasic potassium phosphate, 0.5% sodium chloride, 0.6% sodium phosphate dibasic, 1 M magnesium sulfate) 10% LB and 1% cholesterol. Briefly, an overnight culture of *P*. *aeruginosa* PAO1 was diluted in LB broth to obtain an OD600 of 0.1 in the presence or absence of 1% CBO, and incubated at 37 °C for 24 hours. Cells were pelleted by centrifuging at 10,000 × g for 5 minutes, washed free of the CBO by rinsing three times with minimal media (M9), and then resuspended in M9 (OD600 = 0.1), prior to introduction to *C*. *elegans*. When the larvae were grown up to L4-young adult stage, nematodes were infected with *P*. *aeruginosa* (10^8^ CFU/ml). Mortality was recorded daily for 4 days. Nematodes that did not display movement in the microtiter plates were considered dead. Uninfected *C*. *elegans* with *E*. *coli* OP50 was used as the control. Infections were performed in triplicate with four replicates per experiment. Survival curves were plotted by Kaplan and Meier survival plot using Graph Pad Prism.

### Extraction of AHL

*P*. *aeruginosa* PAO1 culture was grown at 30 °C with shaking at 150 rpm in 100 ml LB liquid medium. After incubation for 18 h, the culture was centrifuged at 6,000 × *g* for 10 minutes and the supernatant was obtained for extraction. An equal volume of acidified ethyl acetate (0.01% v/v glacial acetic acid in ethyl acetate 7:3) was added to the supernatant and the organic phase was separated by centrifugation (10,800 × *g* for 15 minutes at 4 °C). The extracts were evaporated to dryness using a rotary evaporator and stored at −20 °C for further analysis by LC-MS.

### Effect of exogenous AHLs on violacein Production of *Chromobacterium violaceum*

To study the effect of exogenous AHLs on violacein production, AHLs were extracted from *P*. *aeruginosa* treated with 1% CBO and untreated cultures^48^. These AHLs were added to *C*. *violaceum*, which was pre-treated with 0.1% CBO, to determine effects on violacein production^49^. Violacein was extracted and quantified spectrophotometrically using the method described by Blosser and Gray^50^.

### Targeted quantitation of AHLs by LC-MS/MS

All the MS analysis was carried out on an Agilent 1290 Infinity UHPLC system coupled to an Agilent 6540 UHD Accurate Mass Q-TOF mass spectrometer equipped with a Dual AJS (ESI) source. The samples were introduced to the mass spectrometer through a reversed-phase column (Agilent ZORBAX SB C-18, 2.1 × 30 mm, 3.5 µm) with water containing 0.1% formic acid as mobile phase A and acetonitrile containing 0.1% formic acid as mobile phase B. The flow rate was maintained at 0.2 mL min^−1^. A linear gradient of 5–95% mobile phase B in 25 minutes was used to separate the molecules. Targeted MS/MS data of C4-HSL (protonated mass, 172.0968 Da), C12-HSL (protonated mass, 298.2013 Da) and Leucine enkephalin (protonated mass, 556.2765 Da) were collected by setting the collision energies at 15 V, 15 V and 22 V, respectively. Calibration standards (0.25–250 ng/µl) were prepared using synthetic AHLs. All the samples including the standard preparations were spiked with Leucine enkephalin as internal standard. Infusion of reference masses ensured less than 3 ppm mass accuracy over a wide mass range. Generation of calibration curves and quantitation of the compounds were performed as described^51^.

### Statistical analysis

Statistical analysis was performed using Prism (GraphPad Software Inc., San Diego, CA). Statistical comparisons were carried out using the One-way Analysis of Variance followed by Dunnett’s test and student’s t-test. A value of p < 0.05 was considered significant.

### Data availability statement

The data generated and analyzed during the current study is available from the corresponding author on reasonable request.
